# Ovarian carcinoma associated with pregnancy: A clinicopathologic analysis of 23 cases and review of the literature

**DOI:** 10.1186/1471-2393-8-3

**Published:** 2008-01-20

**Authors:** Nadereh Behtash, Mojgan Karimi Zarchi, Mitra Modares Gilani, Fatemeh Ghaemmaghami, Azamsadat Mousavi, Fahimeh Ghotbizadeh

**Affiliations:** 1 Gynecology-Oncology Department, Vali-e-Asr Hospital, Imam Khomeini Hospital, Keshavarz Blvd., Tehran 14194, Iran; 2 Assistant Professor, Gynecologic Oncology Fellowship from Tehran University Of Medical Science, Shahid Sadoughi University Of Medical Science, Shahid Sadoughi Hospital, Yazd, Iran; 3 Gynecologist Oncologist, Tehran University of Medical Sciences, Tehran, Iran

## Abstract

**Background:**

The aim of this study was to analyze and describe cases of ovarian cancer in pregnant women treated at our center and to review the literature concerned, and to discuss the rationale for therapy.

**Methods:**

Twenty-Three patients of ovarian malignancies during pregnancy were treated at Vali- Asr Hospital between 1991 and 2002. Data on treatment and follow-up were evaluated.

**Results:**

The incidence of ovarian carcinoma associated with pregnancy in our series was 0.083/1000 deliveries. Eleven (47.8%) were found with ovarian malignant germ cell tumors, five (21.7%) with low malignant potential tumors, four (17.4%) with invasive epithelial tumors, and three (13%) with sex cord stromal tumors. Seventeen (73.9%) of the patients were diagnosed in stage I and had complete remission. Five of the six in advanced stage died. The mean follow-up was 36.3 months. The prognosis was significantly related with stage and histological type (*P *< 0.05). Sixteen healthy live babies were recorded in this group, and two premature newborn died of respiratory distress syndrome. Chemotherapy was administered to 44% of the patients, in two cases during pregnancy. Overall survival at 5 years was 61%. In most of case conservative surgical treatment could be performed with adequate staging and debulking.

**Conclusion:**

Early finding of ascitis by ultrasound and persistent large ovarian mass during pregnancy may be related to malignancy and advanced stage. Pregnant women in advanced stage of ovarian cancer seem to have poor prognosis.

## Background

Ovarian cancer is the second most frequent gynecological cancer complicating pregnancy except for cervical carcinoma. Although the overall incidence of ovarian cancer is very low (one in 12500–25000 pregnancies), the routine use of ultrasound in pregnancy has led to more frequent findings of adnexal masses, making diagnosis and management increasingly challenging. The estimated incidence of ovarian tumors is approximately one in 1000 pregnancies. Of those tumors approximately 3–6% is malignant [1-4]. These tumors are relatively asymptomatic and could be seen in a routine ultrasonographic scan [5]. In the absence of large prospective randomized trials and cohort studies, it is difficult to know how best to manage these patients. In dealing with a pregnant woman with ovarian cancer, one must consider the effects of the malignancy on the woman and the fetus and how the pregnancy itself can change the diagnostic procedures and therapy.

Surgical treatment is the same as that in nonpregnant patients. Further surgical treatment depends on the stage, type, and presence of the metastatic pathway [6-8]. This study is a retrospective review of our experience of malignant ovarian tumors diagnosed in pregnancy and treated in our center between 1991 and 2002.

## Methods

This is a clinical history retrospective review of patients with ovarian cancer during pregnancy who were diagnosed and treated at Vali-e- Asr Gynecologic oncology department during a 11-year period between 1991 and 2002. 607 cases of ovarian cancer were treated in this center during 11 year-period. Twenty-three of them were detected during gestation, at delivery, or in the puerperium. Due to our Clinic is a referral center, Eighteen of them were from our obstetrics department, while 227500 deliveries were done in our obstetric clinic during the same period. The others were transferred from other hospitals. Of these patients that were referred, only patients were selected for come to study that surgical staging for these to be done. Specialized pathologists in our institution reviewed pathology specimens and biopsies for all 23 patients. The 1988 FIGO Staging System was retrospectively assigned for each case after reviewing their operative reports and microscopy slides. All patients with the diagnosis of ovarian immature teratomas were histologically graded according to the criteria of Norris. No patient had positive past medical history or any relative family history. Patients were followed up with clinical, tumor marker, and radiologic assessments. Follow-up information was recorded up to the date of last contact or death. Responses to the treatment were evaulated using World Health Organization criteria. Complete response or cure was defined as complete resolution of all clinically and radiologically measurable diseases.

## Results

The age of the patients ranges from 20 to 40 years (with a median range of: 29.2 years). In this group, histopathologic characteristics included malignant germ cell tumors (*n *= 11), malignant epithelial ovarian tumors (*n *= 4), low malignant potential (LMP) tumors (*n *= 5), and sex cord-stromal tumors (SCT) (*n *= 3). Seventeen patients (73.9%) were in stage I, 5 two (8.7%) in stage II, three (13 %) in stage III, and one (4.3%) in stage IV. The clinical and pathologic profiles of 23 patients were shown in Table 1.

**Table 1:** Characteristics of patients with ovarian tumors during pregnancy

**Histological type**	**EOC**	**LMPT(BOT)**	**MOGCT**	**SCST**
No. of patients	4 (17.4%)	5 (21.7%)	11 (47.8%)	3(13%)

Stage I	1	5	8	3

Stage>I	Stage III(2) Stage IV(1)	-	Stage II(1) Stage III(1)	-

Adjuvant chemotherapy	3/4	-	6/11	1/3

Chemotherapy during Pregnancy	1/3	-	1/6	-

Recurrence	3 DOD = 3 Stage III(2) Stage IV(1)	-	1 DOD = 1 Stage III [(IMT(grade 3)]	1(JGCT)

EOC: epithelial ovarian cancer; LMPT: Low malignant potential tumor; BOT: Borderline ovarian tumor; MOGCT: malignant ovarian germ cell tumors; SCST: sex cord-stromal tumors. DOD: Death of disease ; IMT: Immature teratoma; JGCT: Juvenile granulose cell tumor

The most common of patient's initial symptom was pelvic or adnexal mass (11/23, 47.8%). These masses were detected accidentally by routine pelvic or ultrasound examinations. Mean of ovarian size in these were 8.5 cm. Three patients (13.6%) presented with abdominal pain, and seven (30.4%) with swelling and ascites. Two (8.7%) patients were asymptomatic and found tumors in operation accidentally, of which one malignant papillary serous tumor and another dysgerminoma was diagnosed in laparotomy for cesarean section. Five of the seven patients with ascites were in advanced stage (stages III-IV) and died finally. Another two patients with negative cytology of ascites had dysgerminoma and immature teratoma and were free of disease at the last follow-up.

Twenty-two patients were diagnosed during pregnancy, and one in the puerperium. Table 2 presents the allocation and outcomes of patients according to duration of pregnancy at diagnosis. Six were found in the first trimester, and two of them had abortion. One of these who was diagnosed with disseminated ovarian endometrioid cancer at 6 weeks died within 12 months, though she had accepted aggressive cytoreductive surgery and adjuvant chemotherapy. In two women, tumors were found by prenatal examinations at 13 weeks of gestation, and both undertook observations until their terms. The former was with stage Ia serous borderline tumor and got free of disease without additional treatment. The latter that were diagnosed with stage Ia dysgerminoma in operation. Another woman with serous borderline tumor was found at 14 weeks of gestation. She had operation preserving pregnancy at 14 weeks of gestation and got a satisfactory outcome for mother and fetus. Ten gravidas were detected in the second trimester, and all accepted instant operations preserving pregnancy respectively. Exclude five patients that had preterm labor, no perioperative complication was observed in these patients having conservative surgeries during the second trimester.

**Table 2:** Ovarian malignancy identification according to duration of pregnancy and their outcomes

**Stage of pregnancy**	**Number of patients**	**Outcomes of patients**		**Outcomes of pregnancy**	
		
		**DFS**	**Dead**	**Vaginal deliveries**	**CS**
Trimester 1	6	5	1	4	0
Trimester 2	10	7	3	5	1
Trimester 3	6	5	1	2	3
Puerperium	1	1	0	1	0

DFS: Disease free survival; CS: Cesarean section

Four patients underwent Cesarean sections and immediate surgery for tumor directly after detection in the third trimester, of which three with early-stage malignancies had satisfactory outcomes and one in stage III c died. Another one gravidas accepted 11-week observation awaiting fetal maturity before their aggressive surgeries. She had Disgerminoma stage Ia and got complete remission. One with endodermal sinus tumor (EST) stage I received chemotherapy and got complete remission. One patient in the puerperium had immediate surgery for tumor with stage II disgerminoma that received chemotherapy and had complete remission.

All patients undertook surgery as the initial treatment. Surgery varied from biopsy to total abdominal hysterectomy and unilateral or bilateral salpingo-oophorectomy, omentectomy, and debulking. In young women interested in further fertility and with LMP tumors and early-stage germ cell tumors, surgery was conservative. Two cases of advanced cancer were treated by primary cytoreductive surgery. Two cases underwent secondary debulking. In 10 cases (43.7%), additional chemotherapies were administered. Two patients received chemotherapy with the fetus in utero. One patients with papillary serous adenocarcinoma stage IIIc that received 4 course of taxol plus carboplatin (T+C) from 20 weeks of gestation. she underwent Cesarean section and optimal debulking in 37 weeks of gestation. After termination of pregnancy she received 3 course of T+C and 3 course of interaperitoneal carboplatin for consolidation therapy.4 months after she was recurred and received second line chemotherapy. She died 38 months after. Another patient with immature teratoma grade 3, stage III that received 2 course of bleomycin, etoposide and cisplatin (BEP) from 29 weeks of gestation. These patients had good pregnancy outcome but all of these died.

The mean follow-up was 36 months (range, 12–73 months). In this period the patients followed with measurement of CA125 level, ultrasonography and Computed tomography if to be indication. The overall expected 5-year survival was 61%. Invasive epithelial cancer had the poorest outcome in all histologic types. All patients in early stage except the one lost were free of disease, whereas 80% of the five in advanced stage died within 2 years and all of them died within half year. Of the four patients in advanced disease, three were with invasive epithelial cancers, and one with immature teratoma. All three patients with epithelial cancers died.

No congenital malformations were detected in all 16 newborns. Sixteen healthy term infants were delivered by twenties normal vaginal births and four Cesarean sections. Other five premature newborns were born by normal vaginal births, of which two died of respiratory distress syndrome due to delivered before 26 weeks. Neither dystocia nor tumor metastasis to placenta and fetus was recorded.

## Discussion

Fewer than 20% of epithelial ovarian cancers occur in premenopausal women. However, now that ultrasound monitoring is routinely used during pregnancy, adnexal mass findings in pregnant women are relatively common. Ovarian cancer is the second most frequent gynecologic cancer complicating pregnancy [1-3].

Incidence of ovarian malignancies detected during pregnancy was 1/15000 to 1/32000 pregnancies in most reports [3-9]. Ueda and Ueki reported a higher incidence with 1/1684 pregnancies [6], but population-selective bias was not excluded in their study. In our series, carcinoma of the ovary during pregnancy remains a rare event and occurs in 0.083/1000 deliveries. Similar incidence as 0.08/1000 deliveries has been recently published in Sayedur's study and PUMCH[1,4]. In the most numerous and most recently informed series by Leiserowitz [10], with 202 cases of ovarian cancer in pregnant women, there is a 0.19% proportion of ovarian mass diagnosed during pregnancy, of which 2.15% were cases of ovarian cancer.

Our method and results were similar to Zhao et al. [3]. Most of our patients were clinically asymptomatic at the time of presentation. Such phenomenon has been described previously [7,8]. Ovarian tumors are estimated to occur in 1 in 81 pregnancies to 1 in 2489 pregnancies, and of these 2–5% are malignant [3,4,8-12]. With the widespread use of routine prenatal ultrasound, the finding of an adnexal mass in pregnancy is an increasingly common occurrence [4,13]. Furthermore, our data reveals that the early finding of ascites by ultrasound may be closely related with malignancy and advanced stage and bad prognosis. This implication previously investigated by Zanotti, et al. [4].

The majority of ovarian cancers associated with pregnancy are diagnosed at an early stage, when disease is still confined to the ovary [4,14]. It seems that the distribution of different histologic types of ovarian cancers during pregnancy is similar to that of nonpregnant women in the corresponding reproductive-age group (Table 3)[4,14-16]. In our series, germ cell tumors and epithelial tumors of LMP were much more prevalent than other types of malignancies in pregnancy. The most common subtype of germ cell tumor in our study was disgerminoma, and next were immature teratoma and EST, which are similar to another reportes [11-18]. Reflecting trends in the reproductive-age population, the majority of ovarian epithelial tumors diagnosed during pregnancy are tumors of LMP and most are confined to the ovary [12,14]. Granulose cell tumor is the most common subtype of ovarian cancers derived from the specialized gonadal stroma, presenting as the juvenile form in young females[19]. Similar events have been seen in our group and Zanotti [4]. In the largest reported series of SCTs diagnosed during pregnancy, two of their patients were associated with virilization, and one with vaginal bleeding [19]. Masculine signs in pregnancy were present in two of five patients with SCT in Duska's report [20] but were absent in Gurbuz's case [21]. Young *et al*. [19] have revealed the biologic behavior of SCTs diagnosed during gestations was similar to that of tumors unassociated with pregnancy. Thus, complicating gestations could not make worse the prognosis of ovarian malignancies if adequate treatments were applied in time, but in our series one patient with juvenile granulose cell tumor was multiple recurred and died 48 months after.

**Table 3:** Allocation of ovarian in pregnancy by histology of tumor in literature

	**Author/year**				
**Histopathology**	**Dgani/1989**^(12)^	**Copeland/1996**^(13)^	**Zanotti/2000**^(2)^	**PUMCH/2003**	**This study**
	
Epithelial malignancies	65%(15/12)	37.5%	33–40%	50%(11/22)	39.1%
LMP	35%(8/23)	-	2/3	27.3%(6/22)	21.7%

Invasive epithelial	30%(7/23)	-	1/3	22.7%(5/22)	17.4%

Germ cell malignancies	17%(4/23)	45%	30–33%	40.9%(9/22)	47.8%

SCTs	13%(3/23)	10%	17–20%	9.1%(2/22)	13%

Others	5%(1/23)	7.5%	12–13%	0.(0/22)	0

LMP : Low malignant potential ; SCT: sex cord-stromal tumors

There were reports about the rapid growth and recurrence of ovarian germ cell tumors during pregnancy[4,22,36]. Most of these reported cases got satisfactory results after standard postoperative chemotherapy. Mooney *et al*. [23] Described multiple areas of microinvasion in eight of ten reported serous tumors of LMP diagnosed during pregnancy. However, these aggressive features seemed to regress with termination of the pregnancy, and all ten cases got free of disease. No such invasive behaviors were found in our series. Obstetric complications were numerous in the studies of Karlen *et al. *[22] and Bakri *et al*. [11], who noted that 47–50% of patients had associated complications such as Cesarean section and observed that the management of pregnancy was frequently altered to accommodate for the presence of the ovarian tumor. Adverse effect on the fetus was demonstrated by the 0–24% fetal death rate presumably attributable to alteration in the obstetric management [11,22]. Our data show two(8.6%) premature fetuses died of respiratory distress syndrome. Zemlickis *et al*. [24] documented that maternal cancer probably caused suboptimal intrauterine conditions and increased risk of stillbirth (a 4.23 relative risk) in women who did not receive chemotherapy. The higher overall survival rates in our series were attributed to 78.3% of patients in stage I of the disease and more patients with germ cell tumors and tumors of LMP. But Overall 5-years survival was 61%. Advanced stage of disease and special histologic type, especially invasive epithelial cancer, were the important poor prognostic factors [24-28]. Unilateral, simple-appearing masses less than 5 cm in diameter detected in the first trimester often represent cysts that are functional in nature. For an adnexal mass exceeding 6 cm, with complex structure or ascites or persisting 16 gestational weeks, surgical intervention is important to obtain a final histologic diagnosis and rule out malignancy [26]). Elective surgery for tumors with low suspicion of malignancy should be delayed until the second trimester (17–19 weeks of gestation), a time associated with a reduced risk of spontaneous abortion, hormonal independence of the corpus luteum of pregnancy, and resolution of functional cysts in the vast majority of cases[27,28]. Our experience shows adnexal surgery in the second trimester is safe for mother and fetus. We had 8.7% abortion and 70% of patients had full term pregnancy.

The spontaneous abortion rate after surgery in the first trimester was documented as 10%, 76.3% patients progressing to full-term delivery [29]. Hysterectomy during pregnancy is rarely indicated, unless it contributes significantly to tumor debulking [4,13]. As follows, we summarized our therapeutic experience and others according to histologic types.

LMP tumors differ from invasive epithelial ovarian cancer in their indolent behavior and good prognosis. In our study, all five LMP tumor patients had no evidence of disease at the end of follow-up whether they received aggressive surgery and postoperative chemotherapy or not. All six cases in Gotlieb's report[30] with immediate conservative surgery preserving pregnancy had satisfactory outcomes. Since there was no established benefit from postoperative therapy for tumors of LMP even in late-stage diseases[31] and most recurrences were still borderline [30-35], adjuvant chemotherapy should not be dictated especially for cases in pregnancy. Invasive epithelial cancer has the worst prognosis in all types of ovarian cancers. In our Experience, none of the three with advanced disease survived. Contrarily, one well differentiated stage I patients were still alive. All had postoperative platinum-based 12 chemotherapy and in one patients combination chemotherapy had done during pregnancy. For these cancers, timely cytoreduction surgeries should be applied, and postoperative adjuvant chemotherapy is indicated, except for well-differentiated stage IA tumors[6]. It should be stressed that chemotherapy is contraindicated during the first trimester of pregnancy because of the high rate of abortion[22,36] and abnormal fetal development[39], whereas it is compatible in the second or third trimester when the risk of congenital malformation for fetuses exposed to chemotherapy is no greater than the general population[24,37]. However, there are non teratogenic effects of chemotherapy such as intrauterine growth restriction (low birth weight) or effects on the central nervous system as it develops throughout pregnancy[24,38-40]. Until now, no studies have evaluated the long-term consequences for children exposed to intrauterine chemotherapy. Breastfeeding during cytotoxic chemotherapy has been discouraged in general [39-42]. There is no convincing evidence that a synergistic increase in malformations occurs with the use of multiagent regimens as opposed to treatment with a single cytotoxic agent [38]. The literature contains numerous reports of bleomycin, cisplatin, and etoposide used in pregnancy with no untoward effects [3,41-44]. Several reported cases in the literature has described the use of adjuvant cisplatin and cyclophosphamide initiated in the second trimester of pregnancy, all with good response to therapy and subsequent delivery of a healthy fetus [43-46]. There were few case reports describing the combined use of paclitaxel and carboplatin in human pregnancy, and there seems to be no significant fetal toxicity when administered during the second or third trimester [46-49].

## Conclusion

Early finding of ascitis by ultrasound and persistent large ovarian mass during pregnancy may be related to malignancy and advanced stage. Pregnant women in advanced stage of ovarian cancer seem to have poor prognosis. Chemotherapy is not contraindicated during the second or third trimester, but the choice of couple must be considered.

## Competing interests

The author(s) declare that they have no competing interests.

## Authors' contributions

NB: conception and exiting of manuscript.

MKZ: Literature search and preparation of manuscript.

Other authors: done surgery of patients and help for writing the manuscript.

All authors read and approved the final manuscript.

## Pre-publication history

The pre-publication history for this paper can be accessed here:


